# Community crime prevention in Portugal: an introduction to Local Safety Contracts

**DOI:** 10.1057/s41300-021-00112-2

**Published:** 2021-03-27

**Authors:** Ana Amante, Miguel Saraiva, Teresa Sá Marques

**Affiliations:** CEGOT—Centre of Studies in Geography and Spatial Planning, of the Faculty of Arts and Humanities, University of Porto, Via Panorâmica s/n, 4150-564 Porto, Portugal

**Keywords:** Crime prevention, Local Safety Contracts, Proximity Policing, Multidisciplinary approach, Portugal

## Abstract

Following the philosophy of other international programs as proximity policing or situational crime prevention, the Local Safety Contracts (CLS) have been an innovative strategy in Portugal, as they allow the sharing of accountabilities between the central and the local administration, in association with the police and the community. Nonetheless, little has been written in Portugal about such strategies, and nothing at all for the international scientific community. The aim of this paper is therefore to present the CLS, discussing their crime prevention stance and their impacts on local communities. First, the new preventive and multidisciplinary organizational model that is at the basis of CLS is discussed. Then a qualitative assessment of implementation is made through a set of interviews to relevant actors. Conclusions are drawn based on the experiences of municipalities, police and administration, contributing to the debate on community crime prevention, and enhancing the need for multidisciplinary, multilevel and place-specific approaches.

## Introduction

Preventing crime and reducing insecurity have never stopped being core goals of modern societies. Today, the most important policy agendas concerning quality-of-life, sustainable development or territorial cohesion (EC 2017; OCDE 2020) explicitly state the importance of devising strategies for increasing urban and human safety. After the economic crisis of the last decade, and now with the pandemic of COVID-19, social and territorial vulnerabilities have been exacerbated, originating increased impacts that are not equally distributed, as they often depend on local contexts (Marques et al. 2019; Sucic and Karlovic 2017; Artelis 2017; Killias 2000). Furthermore, despite the widespread decline in global crime (Farrell et al. 2014), also found in Europe and Portugal (SSI 2019), particular phenomena have increased, such as “crimes against people” or “crimes against society” (Eurostat 2020)—including against the elderly or woman—which some authors relate to recent economic, social and political unrest (Vieno 2013).

Thus, territorial planning needs to address prevention strategies both at macro- and micro-levels (DGT 2018). These strategies should be framed by alternative paradigms of security (Canhoto 2010; Oneto 2019), that is, they should integrate models of preventive policing (Fernandes 2015), models of community participation (Loveday 2018; Saraiva et al. 2016), and advances in environmental criminology which state the importance of place-specific interventions in social, urban and geographical components as key instruments of prevention (Andresen 2014; Weisburd et al. 2016; Wortley and Townsley 2016; Saraiva et al. 2019).

Over the past decades, the focus of this field has shifted from the deviant characteristics of individuals who commit crimes, to a more complex view of the crime phenomenon (Wood and Shearing 2007; Weisburd et al. 2010; Leitner 2013). In countries like the USA, England or France, models of security and social control were replaced by concepts of policing based on local strategies and public participation. This accompanied a “smarter planning” paradigm, geared towards the pursuit of political and social equality where citizens could play an active role in the decision-making process on public policies (Davidoff 2007). Inclusion is deepened by fostering public debate and promoting new citizenship processes, where each individual is heard and is given the ability to intervene (Allmendinger 2017).

This way of thinking led to the development of several models of policing over the last three decades, whose pertinence is still very much at the fore of research and implementation, regardless of country and political interests (Bolas 2019). These include Community Policing (Trojanowicz and Bucqueroux 1998; Bayley 1996; Fisher-Stewart 2007; Hope 1995; Lawrence and McCarthy 2013; Gill et al. 2014); Problem-Oriented Policing (Goldstein 1990; Weisburd et al 2008; Ward 1998; Braga 2008); Situational Crime Prevention (Sutton et al. 2008; Rosenbaum et al. 1998) and Proximity Policing (Brien 2015; Bolle 1998; Casey 2010; Monjardet 1996; Jenkins 2013). All these models have in common sharing the accountabilities between different actors and the community, although they differ in terms of institutional structures, which are also variable from country to country, influencing the way they are implemented.

Theories such as Situational Crime Prevention (Clarke 1980), Routine Activity Theory (Cohen and Felson 1979) and Rational Choice Theory (Clarke and Cornish 1985) have shifted the focus from traditional criminology approaches, particularly those based on the characteristics of offenders. Situational Crime Prevention argues that prevention strategies should be focused on existing problems and understood at the microscale. Hence, these depend on contextual situations and must be defined together with stakeholders in order to balance public safety, individual rights, community concerns and other factors such as economic investments and the capacity for implementation. Being strategies based on action and on the reduction of opportunities, they relate to a Problem-Oriented policing perspective (Goldstein 1990), which provide police organizations with more knowledge on different implementation tools to respond to criminal, but also physical and social problems.

Precisely, both community policing and proximity policing models relate to Situational prevention, as they implement a succession of preventive strategies that support changes in social conditions (Hope 1995; Rosenbaum 1988). According to Rosenbaum (1988), community crime prevention is used as a holistic concept, insofar as the approaches adopted have an impact on the community as well as in their specific urban contexts. This means that the models have moved towards sharing and shifting police responsibilities to other public and private entities (Joshua and Graeme 2017). Although the concept of community policing has several interpretations in the literature (Trojanowicz and Bucqueroux 1998), there are common theoretical principles and strategic frameworks, which include the engagement of the community and community-partnerships with police departments in solving crime and social disorder (Fisher-Stewart 2007).

In the particular case of Portugal, security forces and communities are considered to have practically the same role in community policing. However, the police officers allocated to this policing model cannot actually deal with criminal problems. These agents are called “municipal police” and are under the tutelage of the local municipalities, which manage the fieldwork and professional training of these agents. On the contrary, despite having more similarities than differences, proximity policing models differ from community policing by including not only this awareness of local problems but also specific security strategies at local level Araújo (2019). Proximity security policies have been implemented for the last three decades, with some special programs as “Safe School” (1992) or “Safe Trade” (1998) achieving large recognition in the community. However, Araújo (2019) differentiates these from a true Proximity Policing philosophy that came to prominence in the 21st Century, particularly in Lisbon where its Municipal Police shifted towards a proximity policing program in 2007 (Diniz 2011). This resulted from the administrative decentralization the country witnessed in several sectorial domains, but also from the new Integrated Model of Proximity Policing, approved by the Public Safety Police in 2006 (DNPSP 2006). Hence, this approach of crime prevention is moving towards a multi-scale and multilevel paradigm, following the country’s legislative framework.

As a direct consequence, the local safety contracts (CLS) came to being in 2009, as a new paradigm of proximity security in Portugal, where knowledge and processes of proximity intervention and proximity policing were entwined with proximity safety policies, thus merging various spheres and levels of decision-making. By readapting several models of governance, among different actors and institutions, this paradigm shift enabled the development of territorial instruments specifically focused on urban safety, closely associated with planning development guidelines. These policies have been based on crime prevention and social cohesion strategies and have been implemented through a large spectrum of action and social control initiatives. As Oneto (2019) notes, a model of proximity, prevention and social support to the most vulnerable population has been followed, based on (i) an interpersonal contact, attentive to communities and their problems; (ii) a relationship of trust between communities and police officers, maintained and nurtured over time; and (iii) a multidisciplinary complicity, for working together, solving problems and caring for the community.

Consequently, CLS have quickly become a pioneering example of preventive practices in Portugal, by combining the proximity policing model with the new organizational model for sharing responsibilities between different levels of governance, particularly after governmental decentralization and the new competences assigned to local security forces and municipalities. CLS have thus been an aggregating instrument (Oneto 2019) of the interests of local administrations, policing organizations and national public policies.

This paper aims to introduce the Portuguese CLS to an international scientific community, by summarizing the main stages of their development, from conception to implementation. Section 2 presents the origin of CLS, how they fit into the Portuguese paradigm shift towards prevention practices, and their implementation model. Section 3 describes the research method. Section 4 summarizes a series of speeches and interviews, describing the involvement of different actors—local administration, central administration and police—in the implementation of CLS. Finally, Sect. 5 presents the conclusions.

## CLS: Portuguese paradigm shift

### Background

Portugal, a small country located in Western Europe with 10 million inhabitants, is today considered one of the world’s safest with one of the lowest victimization rates in Europe, being in third place in the Global Peace Index (IEP 2019). The paradigm shift started in 1999, when the Public Safety Police (PSP), under the tutelage of the Ministry of Internal Administration (MAI), moved from a military to a more civil model (Law 5/1999). However, until 2006, it can be said that the reactive model carried out in Portugal theoretically privileged just two of the four main pillars of policing: rapid response to incidents and criminal investigation (the other being information and criminal prevention) (Guinote 2019).

This model was originally developed in the first half of the nineteenth century, for the control of incidents, the preservation of evidence at the crime scene and for the collection of technically more sustained evidence to increase convictions in court. Such a model was maintained well until the 1980s, as the State was considered in Portugal as the single political entity, whose priorities were perhaps not aligned with those of the population. National security was mostly oriented towards external threats and based on a horizontal structure of competences. The post-revolutionary society (Portugal’s authoritarian regime ended in 1974) began to differentiate itself from what was the state's priority. Concepts as Societal Security (Waever 2008) led to a so-called philosophy of proximity in Portugal (Guinote 2019), where National Security was differentiated from other problems related to the internal presence of vulnerable groups. As a consequence, a reactive model based on the premise of power (the purpose of the police was to fight crime) was increasingly replaced by a proximity and preventing model.

Until 2006, this was mostly a “community policing” model, based on a skill-sharing paradigm between citizens and police. The first forays into this model were experienced in the 1990s, such as the previously mentioned “Safe School Program”, devised to tackled increasing outbreaks of conflict in schools. This was the first programme implemented by the PSP and also the first application of the concept of Societal Security. Later, an approach more focused on trust-sharing and regular contacts between police and community—i.e. a model of “proximity”—started to be implemented. This was based on several international experiences, as Scotland’s *Team Policing*; England’s *Unit Beat Policing*, *Neighborhood Team Policing* or *Crime Prevention Units*; France’s *Police de Proximité*, as well as other examples from the Netherlands and Belgium (David 2014; Guinote 2019). These models were replicated fitting the framework of the Portuguese legislation, through special programs promoted by MAI. Also, by 2004 the Municipal Police was created in Portugal (Law 19/2004 of 20th May) as an instrument to territorialize safety and increase the connections to the community (Diniz 2011).

Finally, in 2006, within the scope of the administrative decentralization process, Strategic Directive No. 10/2006 of May 15 presented a legislation shift that allowed the creation of the Integrated Proximity Policing Program (PIPP). With it, urban security ceased to be a sectoral issue to become a cross-cutting, multilevel theme in planning and prevention. The resulting shift, along with the new Law of Internal Security two years later (no. 53/2008), led to reconfiguration of the competences for safety between the Central Government, the Local Administration and security organizations. Although the State, through the Ministry of Internal Administration, maintained responsibility for security in the country, these laws gave more preventive and proximity capacity to local organizations and to the training of agents, and empowered the communities. The strategic main lines of this programme were to create patrolling teams that could ensure a personalized relationship to communities and urban areas, thus building trust that could help to collect essential information about the problems and their causes, manage incidents, and contribute to prevention.

In the following years, some proximity projects were implemented, with Lisbon serving as a test ground for the rest of the country. PIIP has since been renamed the Integrated Proximity Policing Model (MIPP), and includes more than 1.000 Portuguese agents in its ranks. It was in this context that CLS, a nation-wide programme, came to being. By combining interests from the various spheres of central and local administrations, CLS were perceived as guiding instruments of public policies, focused on implementing crime prevention strategies through a proximity policing model. A trial experience starting in 2008 became inactive shortly after due to the change in Government and the global economic crises that severely affected Portugal (Teixeira 2018). A second, more prominent generation started in 2016.

### Organizational structure and the new generation of CLS

In 2016, a new generation of CLS was presented, accompanying the process of administrative decentralization in the country and a greater articulation of the Internal Administration with local powers to enhance territorial and social cohesion. Municipalities were empowered, having an increased role in coordinating operational resources, performing safety diagnosis and implementing solutions. However, this changing organizational model also allowed other entities in the three levels of intervention (political, coordination and operational) to share accountability. Such configuration of competences was officialised in July of 2016, with the signing of a Cooperation Agreement. In addition to the Central and the Local administration, other governmental entities were included as the Ministries of Citizenship and Equality; Justice; Science, Technology and Higher Education; Education; Employment, Solidarity and Social Security; Health; and associations as the Association of Portuguese Municipalities and the National Association of Parishes.

**Fig. 1:**
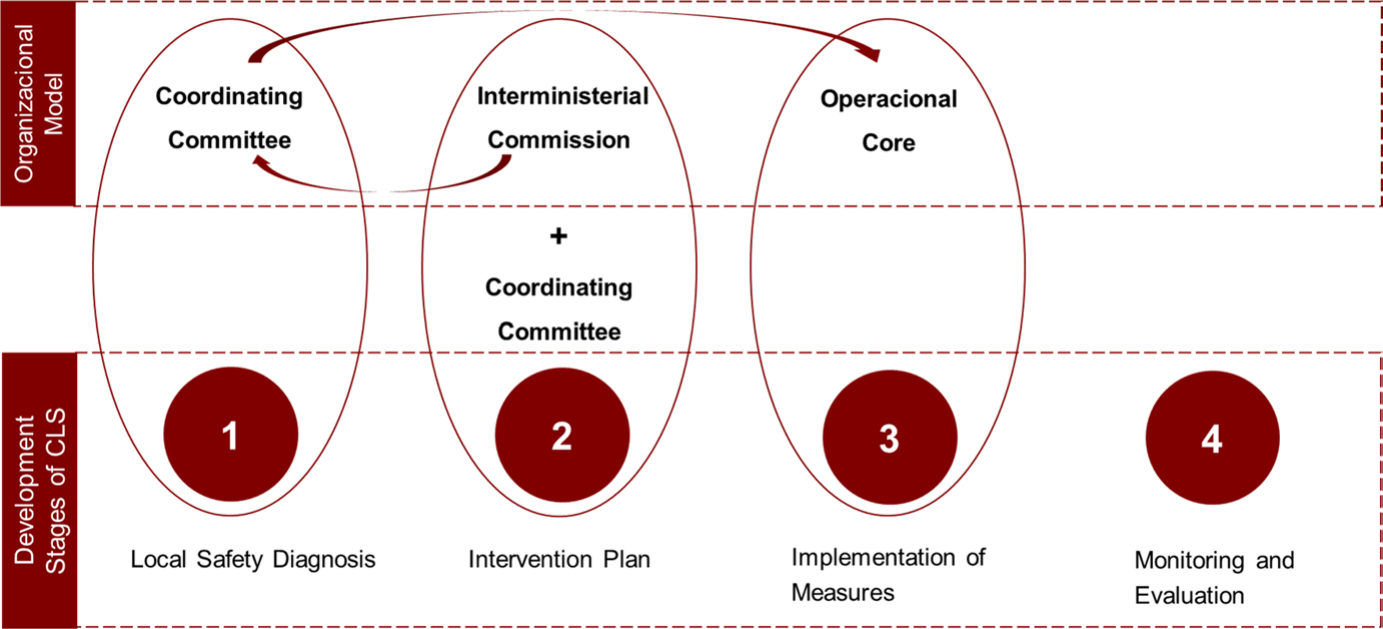
New organizational model and stages of CLS ( source: authors)

This organizational model was divided into three levels, fulfilling different purposes; the Interministerial Commission, the Coordinating Committee and the Operational Centre (Fig. 1). The Interministerial Commission is comprised by the above cited ministerial entities. It monitors the actions implemented in the territories through the Coordinating Committee. In turn, this Committee, headed by the Mayor and including the security organizations, approves what is done by the Operational Core group. The group is composed by all the decentralized services of the Central Administration, provided by the municipalities to all other local partners that constitute the CLS contract, including the community associations. Together, they develop the guidelines for action defined in the diagnosis and action plan phases. These—the Local Safety Diagnosis and the formulation of the Intervention Plan—are the first two of the four stages of operationalization of CLS (Fig. 1). The other two stages are the Implementation of Measures, and Monitoring and Evaluation. In a broad sense, Local Safety Diagnosis are meant to be prepared by Coordinating Committee of each CLS. Action Plans are meant to be produced together by the Interministerial and the Coordinating Committees. At this stage, types and conditions of partnerships, as well as measures to be implemented and material and financial resources available, are identified. The Operational Core is then responsible for implementing the measures defined in each Intervention Plan.

Based on a principle of institutional cooperation, CLS tackle four major axes of intervention (Table 1): (i) Prevention of juvenile delinquency; (ii) Requalification of urban areas to reduce elements that promote crime, boosting prevention (associated to environmental criminology principles as CPTED); (iii) Reduction of social vulnerabilities; and (iv) the Promotion of citizenship and other relevant stances as gender equality. Table 1 further describes the major guidelines associated to each intervention axes.

**Table 1:** Intervention axes and guiding measures of CLS

Intervention axes	Guiding measures
Prevention of juvenile delinquency	Awareness-raising and supervising actions of road safety with school students
	Social Inclusion through music
	Development of personal and social skills, through sports
	Creation of conflict mediation programs
Requalification of urban areas	Removal of abandoned vehicles
	Removal of Graffiti and development of urban art projects
	Increase the frequency of the cleaning of urban areas
	Setting up facilities for community use (kiosks, playgrounds, …)
	Intervention in urban spaces, including improvement works, gardening work, replacement of mailboxes and of light fixtures
Reduction of social vulnerabilities	Promoting informative and awareness-raising actions on addictive behaviours, domestic violence, dating violence, cyberbullying, healthy eating and oral hygiene, among others
	Development of professional training courses, inclusion courses and EFA courses (adult training and other learning and training courses)
	Development of activities to improve/promote living conditions within communities, addressing the themes of domestic management and organization of daily life
	Inspection actions on olive production farmlands in Serpas’s municipality and raising awareness about “Who can I hire?” and “Trafficking in human beings”
Promotion of citizenship	Film screenings in schools related to human rights and citizenship
	Educational actions promoting citizenship and social participation
	Supporting the regularization of the documents of immigrants (by SEF – Immigration and Borders Service, and local associations)
	Training peer skills and cultural mediation

In order to minimize the diversity of territorial and social responses in terms of crime prevention, i.e. in order to cater to the specificities of each territory, the Ministry of Internal Administration (MAI) created three different categories of CLS: MAI Municipality, MAI Neighbourhood and MAI Citizen. These categories are distinguished by the characteristics of the areas to be intervened. MAI Municipality aims to coordinate public policies for safety at municipal level. MAI Neighbourhood is composed of strategies of social integration and crime prevention, aimed for example at juvenile delinquency or the protection of public space, according to the existence of place-specific social vulnerabilities in given urban areas. Finally, MAI Citizen focuses on areas where atypical and/or specific situations occur which may hinder citizens’ daily lives. Consequently, these are deemed to be interventions relatively restricted in space and time.

**Fig. 2:**
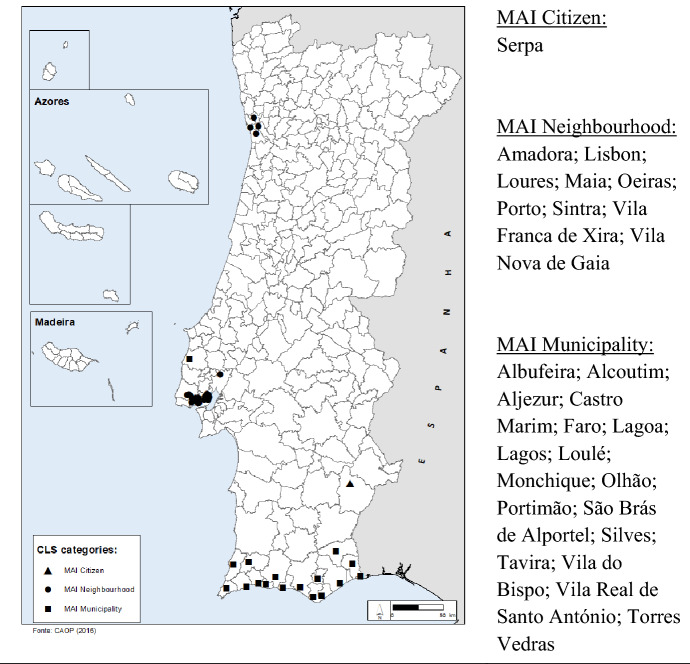
Spatial distribution of CLS in Portugal, by categories ( source: authors)

According to the most recent information, forty-five CLS contracts were celebrated in twenty-seven Portuguese municipalities (9% of the total number of municipalities) between 2016 and the present day. These municipalities are signalled on a map of Portugal in Fig. 2. It is evident that the implementation of CLS has been restricted to specific areas of the country, namely around the city of Porto (in the North; the country’s second city); around the capital city of Lisbon (in the Centre); and the region of Algarve, in the South, where the majority of CLS are located. The typology MAI Neighbourhood has been privileged around Lisbon and Porto, where 9 municipalities signed contracts, whereas 16 of the 17 MAI Municipalities are in the Algarve. The regions of Lisbon, Porto and Algarve are precisely the ones that contain higher number of registered crimes in Portugal. Lisbon and Porto are the areas with the greatest urban and population density in the country, while the Algarve is the prime tourist destination. The only contract that does not fit this geographical profile is also the only MAI Citizen, located in the municipality of Serpa. This is a municipality which at specific times of the year sees the arrival of a large quantity of foreigners from other countries and immigrants, due to the olive picking season.

These contracts are presently at different stages of development. Some are reported to be still in the first stage (Local Safety Diagnosis), while others are preparing their action plans and doing implementation. It is not reported that any of the CLS is yet on the last stage of the process (Monitoring and Evaluation). According to the former Secretary for the Internal Administration, CLS should be carefully developed in stages as they are a time-consuming process, particularly in the early phases of collecting information and diagnosis (Oneto 2019). The former Secretary has recently presented that over 280 initiatives directly associated with CLS have been carried out until 2019; 132 corresponding to the intervention axis Prevention of juvenile delinquency; 24 related to Requalification of urban areas and elimination of criminogenic factors; 73 related to Reduction of social vulnerabilities; and 51 related to Promotion of citizenship and gender equality (Table 1). Overall, 14.352 human resources have been involved in these initiatives, with an investment of 1 million Euros (Oneto 2019).

## Research method

As there hasn’t yet been an overall official evaluation of the CLS, because none has reached its final stage, a decision was made to base the discussions presented on this paper on viewpoints derived from three distinct sources. The first were the speeches and interviews made at a national conference on Urban Safety in 2019. The remainder were interviews and opinions expressed at two workshops on safety, crime prevention and urban planning the authors held with relevant actors from different levels of governance. These include seven mayors or representatives (representing the Local Administration), the now former Secretary of State for the Internal Administration and one of the Ministry’s staff members (representing the Central Administration), and two police chiefs and other agents (representing security organizations).

The guiding framework for the analysis of the interviews and speeches is presented in Table 2. These sessions took place in mid and late 2019. With the overall goal of gaining from the insights of actors from different levels directly involved with CLS implementation, the specific purpose of these interviews was threefold: (a) discussing overall experiences at local level; (b) understanding the key elements for successful implementation; and (c) discussing overall results of the CLS.

**Table 2:** Framework for the interviews

Purpose	
(a) Discussing overall experiences at local level	
(b) Understanding the key elements for successful implementation	
(c) Discussing overall results of the CLS	
*Entities*	*Participants interviewed*
Local Administration	7 mayors or representatives of local authorities (2 from the northern region; 3 from the central region and 3 from the southern region of Portugal)
Security Organizations	2 police chiefs and proximity policing agents
Central Administration	Former Secretary of State for the Internal Administration (MAI) and 1 Ministry’s staff member
*Venues*	
Conference on Urban Safety (Coimbra; February 2019)	
Workshop 1 (University of Porto; April 2019)	
Workshop 2 (University of Porto, December 2019)	
*Main Guiding questions*	
*Main question*: How are CLS being implemented in the municipalities?	
*Other questions*:	
Have CLS shown to be effective in preventing crime? What have been their impacts?	
Is teamwork an important common aspect in the interventions?	
It is possible to verify a common responsibility framework in the actions carried out in the territories?	
What has been done concerning preventive measures?	
How is the relationship of trust with communities achieved?	
How have the community’s insecurities been addressed, in terms of social responses?	
What public space improvements have been made?	

Auscultation relied on semi-structured interviews and Content Analysis techniques (Bardin 2016). The qualitative framework was based on the exploration of realities, representations and opinions of different actors in view of their experiences. Above all, it was important to assess what had been done and understand the expectations of those actors regarding the preventive approaches at the local scale. The structure of the interview was flexible, to allow for the openness of discourses and opinion-sharing. Semi-structured interviews were recorded and fully transcribed, and Content Analysis techniques were applied to identify individual and global standpoints, similarities and differences in discourses, and potentially explanatory factors. Thus, discussion delved on the intuitive and critical knowledge of how each actor assesses their experience within the CLS.

## Viewpoints of different actors

### Mayors’ viewpoint—Local Administration

Mayors make it an issue to highlight that urban safety and prevention now requires shared responsibilities, covering many sectors of governance. They recognize that ensuring urban safety is no longer an exclusive concern of the State, nor of the security organizations. It requires a close relationship between these, at various levels, the community and other organizations; as well as strong links to urban planning, to the management of public facilities, and to the requalification and maintenance of public spaces and buildings. Mayors stated that many territories did not comply with CPTED principles before the CLS program started, which had impacts on how they were perceived.

They further highlight that safety has two facets, one objective and one subjective. Mayors have stated that they are not just concerned about crime rates but also are aware of different types of social vulnerabilities that should be an integral part of crime prevention. They have referred to the non-correlation between crime rates and the feeling of insecurity, and that particular social wants increase insecurity at local level. Consequently, they envisage the reduction of feelings of insecurity and the promotion of equal levels of opportunity as key CLS goals. Hence, it is essential to identify the socio-economic profiles of the communities (Diagnosis) but at the same time allow sufficient time for change to take effect, namely the development of feeling of proximity between security organizations and their communities.

Due to this complexity, it is also essential that CLS Action Plans are implemented based on a systematic approach. For example, intervening in neighbourhoods and urban areas (including improving their accessibility or upgrading their image) has increased the self-esteem of residents and has allowed attracting and accommodating different social strata in the same public space, as different images—images of safety—were provided to communities, inside and outside the neighbourhood.

Discussing specific situations, a Mayor from the Lisbon Metropolitan Area highlighted the issue of juvenile delinquency. In one problematic neighbourhood of the city, repeated offenders began to decrease considerably due to the success of the partnerships developed with local associations. In the Algarve, CLS have had a particular focus on the integration of the Roma community (who live in conditions of extreme poverty) and on housing states (which host disadvantage families). CLS local partnerships promoted a successful on-site program of community participation aimed at discussing and solving particular problems, for example related to the role of women and children in society. At the same time, public space and housing were being rehabilitated and employment policies were being proposed. It is expected that these elements together, under the banner of CLS, culminate in social integration. Precisely, in the northern region of Portugal, the Mayors were keen to highlight how CLS contributed to create more “inclusive” territories. Actions taken have directly impacted on social problems as unemployment, school absenteeism, domestic violence, drug use or isolation of the elderly. They have also included the promotion of active citizenship, through sport and arts, especially among children and youngsters.

Mayors also point out that the most difficult stage is the passage from the Diagnosis to the Plan of Action. Good results in the future are dependent on the allocation of adequate human resources at this stage and of the partnerships established. For example, neighbourhoods which displayed issues related to migrants and other minority groups benefited from a partnership with the High Commission for Migration. This partnership has been deemed a determining factor in successfully including these minorities in CLS programmes.

Overall, Mayors expressed satisfaction in joining the CLS programme, stating as the greatest advantages the ability to create projects based on a wide range of partnerships (covering sports, education, music, arts, and so on), and, most of all, the success of the interaction between proximity policing and the communities. The fact that these projects are part of the same banner—the CLS banner—allows them to be inventoried and monitored by both the municipalities and the central administration. This monitoring has been highlighted as crucial element of the success of the CLS, as it provides support for the development of mechanisms to improve conditions against inequality, school failure and social exclusion, especially among the young.

Nonetheless, Mayors mention that so far the greatest disadvantage has been the lack of monitoring and evaluation of the CLS stages themselves, particularly in terms of execution and results. They are cautious about potential financial losses accumulated, due to the number of actions that need to be promoted by the local partnerships, and that need to have a cyclic continuity over the years. Also, they note that the partners sometimes simply repeat individual actions over time, instead of making them part of a whole. As well, Mayors stress that CLS follow a systemic intervention model, with increased responsibilities for municipalities, but also for all other entities in the consortium. This is just perceived as a disadvantage if all entities do not contribute on an equal footing nor share the information to the same degree between themselves. Finally, Mayors express the wish to have even more community involvement in the future.

### Agents’ viewpoint—Proximity Policing

Police agents first emphasize that despite not being a new concept, proximity policing remains a very pertinent strategy in this day-an-age, extremely suitable to the goals of CLS initiatives. Because societies in general and communities in particular are still very closed onto themselves and self-centred, two main ideas are at the basis of the proximity policing carried out in Portugal: trust and complicity. The role of proximity policing is thus to engage with communities, promoting interpersonal contact, attentive to people and their problems, and oriented towards collecting relevant territorial knowledge. Agents recognize the political interest such strategies evoke, but they also deemed it necessary, due to the several sectors of governance that need to work together for successful implementation. In fact, although they consider that the police is very much involved in the CLS from the early stages, they recognize that this is not just a police project.

The intervention of the police starts by identifying the weaknesses of the territory and of the communities therein. This diagnosis helps to define the actions to be carried out, and the response is triggered by the resources that are already in place, so as to guarantee that the situations are regulated in their initial phase. As such, regular tactical tools are in use, as foot and car patrolling, and information gathering by stimulating the participation of vulnerable communities. However, agents point out different strategies according to different territorial contexts. In larger cities, proximity policing strategies often rely on research and data collection. In low density territories, interventions are often directly aimed at social support, for example by giving support to the elderly that live alone and in very fragile conditions.

The direct involvement of the police in the CLS has also included awareness-raising actions, particularly on driver-oriented road prevention, and on building ties between the police and young people. Pedagogical actions oriented towards youths (such as dressing youngster in uniform and allowing them to take part in traffic stops alongside the agents) are very well received and are deemed to allow them to minimize and manage conflict with other young groups or at home. Urban and social intervention actions also take into account the input of operational agents, who do in-site evaluations. For the past few years, the police has been providing specialized CPTED training course (as those taught at the Lisbon Municipal Police). Such evaluations allow identifying and dealing with local problems in CLS contexts. Like the Mayors, agents also pointed out that juvenile delinquency in some territories was reduced due to the success of these strategies.

Other special initiatives have been carried out by the police in the context of the CLS. These programmes are developed through protocols established with other public entities, for example the above mentioned High Commission for Migration. This specific protocol allows the exchange of technical knowledge and training between the police and representatives of the High Commission, so as to better devise solutions aimed at minority communities and immigrants. This helps the police overcome challenges as the linguistic and cultural interaction and integration. Other successful partnership pointed out by several agents is the above mentioned “Safe School” program. It is the oldest prevention program in the country that is still active and displays a successful partnership between the central administration (namely the Ministry of Internal Administration), the police, and the local school communities.

However, agents have stated that it is hard to manage human resources, making it more difficult to intervene and respond to the challenges of each territory. There are relatively few police agents allocated to proximity policing, and consequently even fewer allocated to CLS initiatives. Agents also reinforce the need to increase the trust between the CLS partners to better manage social problems and implement solutions. Agents recognize that their strategies need to be place-based, hence that these strategies should be adapted to each local community with the aid of local authorities. The capacity to quickly articulate in response to a particular situation is paramount in order to avoid the escalating of conflict situations. Also, some agents discussed that a greater sharing of information between institutions and the police would contribute to avoid social constraints, as domestic violence or problems in schools.

### Administration’s viewpoint—Central Administration

According to the representatives of the Central Administration, the paradigm shift from the reactive to the preventive model of urban security only works if the behaviours and the relationship between the central and local administrations and the security organizations changes as well. The greatest challenge is to properly define the operational and logistical priorities of the hierarchical public entities, from the national to the local scale.

The Ex-Secretary of State has emphasized the great effort that Portuguese municipalities have made to implement national spatial planning and security policies, whilst catering to the specific problems perceived locally. This results in different solutions for different territories. Hence, it is imperative to make an early diagnosis of local problems and their causes; and it should be recognized that the measures implemented according to each axis of intervention (Table 1) cannot be standardized for all the CLS.

Additionally, the representatives of the Central Administration have pointed out the importance of CLS, and of the proximity policing model, in reducing social isolation and discrimination, of the elderly and of the migrant population. In the first case, the long-standing population flow from rural to urban areas has led to situations of isolation and neglect. If social interaction and affective bonds are lost, the situation can get worse, and the efficiency of proximity policing may be hindered. The CLS are seen not only as a way to reduce crime rates, but as a way to instil safety through integration, so new strategies for the protection of the elderly are seen as a priority.

In the second case, related to migrant population and minority communities, integration is also achieved by complementary actions as helping to learn a new language, monitoring working and housing conditions, and supporting the education of children and youngsters. The particular experience of the CLS of Serpa—the only case of a MAI Citizen program in Portugal (Fig. 2)—has shown that the partnership with the Immigration and Borders Service (SEF) was essential for successful implementation of the Strategic Action Plan. On one hand, migrants are made aware of their rights, are warned about labour exploitation and human trafficking, and are helped to regularize their situations. On the other, biases are deconstructed in Portuguese society, by recognizing the role of the migrant in the country’s economy, and striving to integrate them, and their families, in the communities. Nonetheless, it was recognized that greater pro-activity in coordinating the actions and interests of the central and the local administration is needed.

Finally, representatives of the Central Administration also point out the importance of integrating young people in CLS projects. In general terms, often they don’t feel as “belonging to the place”, so actions have been implemented to stimulate bonds between them and their neighbourhoods and communities. This has included intervention initiatives in public spaces where urban art has been used as a catalyst for integration and for opening spaces to other legitimate users. A philosophy has been used of improving public space to develop collective efficacy and sense of place, by empowering young people and stimulating their capacity for action and decision making. In turn, this provides them with useful tools for social integration and the development of their life projects.

## Conclusions

From 2016 onwards, the new generation of Local Safety Contracts (CLS) in Portugal has been the culmination of a slow, two-decade paradigm shift, from a reactive-based to a prevention-based model of policing and crime-reduction. It has accompanied the governmental decentralization of competences, the development of new organizational structures within security organizations, and the adoption of models of proximity policing and local-level strategic partnerships. The CLS have become a pioneering example of empowerment of local authorities, security organizations and communities, who share responsibilities in crime prevention and reduction of feelings of insecurity. It is precisely this sharing of responsibilities that has been praised the most by the actors, and it is also the still insufficient trust, or at least the insufficient ability to collaborate even further, that has been pointed out as something to overcome in the future. Based on the experiences of several actors, it can be said that the CLS have provided major contributions to the Portuguese crime-prevention paradigm.

Firstly, CLS have shown the ever importance of territorial and social contexts. Following the literature on territorial and social cohesion (Marques et al. 2019; Sucic and Karlovic 2017) as well as of environmental criminology (Weisburd et al. 2016), place indeed matters and it defines the solutions to be devised. CLS have called out to the promotion of a *territorial culture* at various levels of governance, but also within the community, that should be able to understand the various geographies of crime problems and act accordingly. These geographies relate not only to the disparate crime rates within the country, but also to dissimilar socio-economic vulnerabilities. Consequently, mechanisms should be devised, capable of monitoring social and territorial inequalities though multivariate and multidisciplinary approaches, and of exploring the relationships between vulnerabilities and the spatialization of planning policies.

Secondly, related to the first, it is important, at lower levels of analysis, to understand how urban elements relate to social, economic and geographic conditions, in order to devise local-based interventions for preventing crime (for example CPTED interventions; Cozens and Love, 2015; Saraiva et al. 2019). Each conditioning element, from the design of public areas to the land-use mix to the presence of specific population groups, may influence, independently or together, feelings of (in)security. Each territory, sometimes at micro-levels such as neighbourhoods or streets, has its own specificities and what can be successfully implemented in one CLS may run the risk of failing in another. As such, a deeper knowledge at the local scale needs to exist, not just of crime statistics but of elements as built design, neighbourhood initiatives and groups, anti-social behaviours, and so on. Sound policies aimed at local problems are deemed to have better results than large-scale strategies.

Thirdly, operationalization should be able to integrate models of preventive policing (Fernandes 2015), participation (Loveday 2018; Saraiva et al. 2016) and governance (Canhoto 2010; Oneto 2019). The central administration is considered to be the main actor in the CLS process, but has to be open to other models of participation and recognize the importance of involving other actors. Several authors as Oneto (2019) or Guinote (2019) have emphasized that the empowerment of local authorities, particularly of municipalities, has become vital for the successful implementation of preventive public policies. But the empowerment of other stakeholders and the civil society is also significant to the whole process, and the efficiency of proximity policing, and hence the effectiveness of CLS, is conditioned by the presence of multidisciplinary and multi-sectorial participation. As the CLS are an instrument of planning, they can and should accommodate several models of governance. Safety should be perceived in a wide sense, also covering perceptions, fears and aspirations. This inclusive dimension of security policies, aligned with urban policies, is central to successful implementation.

Fourthly, CLS strategies have to be “out of the box” and should not come from the top-down. Strategic goals, responsibilities and the solutions present in the Plan of Action must be worked together and shared by all. As stated above, a common empowerment at local level is decisive for implementation. Successful examples of CLS implementation, for example relating to juvenile delinquency, have stemmed from a combination of modifying urban elements, reducing social vulnerabilities (particularly of minority groups) and promoting citizenship and gender equality. As mentioned above, weaknesses have been highlighted related to occasions where articulation within partnerships is not sufficiently developed. The commitment of all in implementing and managing the CLS is necessarily to keep the project in progress. Therefore, continuous networking is regarded as a priority (Oneto 2019).

Fifthly, coming full circle to the first point, policies for crime prevention, policing and integration must be systemic. A multidisciplinary and inter-sectoral approach should be implemented to support the promotion of the territorial dimension of public policies at different scales. Territorial and social cohesion is achieved by considering aspects of territorial governance and organization, by promoting social and territorial solidarity and equity, and by recognizing the need for diversity and specificity in territorial policies (Marques et al 2018). Crime and insecurity have a great impact in communities, at local level, so all stakeholders, from the central administration to the community, should work together devising specific strategies for specific territories. On the other hand, proximity policing should be made an integral part of the National security system.

Finally, according to Lourenço (2019), more CLS contracts were expected to be celebrated with other Portuguese municipalities in the near future. However, these declarations are prior to the pandemic outbreak of COVID-19 in early 2020, with no official information on CLS being released since. It should be reminded that the first attempt at CLS in 2008 was severely hindered by the economic crisis. So, further research is needed to understand the impacts of the pandemic in the execution of the CLS. More than ever, as close physical contacts have been severely hindered, integration between institutions and models of governance to tackle urban and social vulnerabilities, as well as feelings of insecurity, are needed.
